# How do industry and province attributes impact corporate contribution to poverty alleviation: A multilevel analysis

**DOI:** 10.1371/journal.pone.0293505

**Published:** 2023-10-26

**Authors:** Shuhan Chen, Guangqing Yang

**Affiliations:** 1 School of Economics and Management, Fuzhou University, Fuzhou, 350108, China; 2 School of Economics and Management, Minjiang University, Fuzhou, 350108, China; University of Almeria: Universidad de Almeria, SPAIN

## Abstract

This study employs a multilevel model, nesting firm observations within industry and province groups, to investigate the influences on corporate contributions to poverty alleviation while considering the industrial and provincial contexts. Using a sample of Chinese firms listed in Shanghai and Shenzhen Stock Exchanges between 2016 and 2019, we find that Herfindah-Hirschman Index (HHI) does not affect corporate contribution. The results show a significantly negative relationship between industry dynamism and a firm’s substantial poverty contributions, as well as a significantly positive relationship between number of state-owned enterprises (SOEs) in industry and the likelihood and extent of a firm’s contributions. Moreover, a firm’s likelihood to participate in anti-poverty activities and make substantial contributions is affected by more intense government intervention and lower per capita GDP. A province’s poverty rate is positively associated with the extent of corporate investments in poverty alleviation. Additional analyses note that firms competitive in an industry that is less dynamic environment are more likely to invest funds into poverty alleviation instead of material contribution. Moreover, for firms headquartered in an industry with more SOEs and in provinces with a stronger government, a higher poverty rate and lower per capita GDP mean it is more likely for them to make both monetary and material contributions for anti-poverty campaigns.

## Introduction

Global poverty represents one of the most important challenges of our time. According to World Bank data, 9.2% of the world’s population, or 689 million people, live in extreme poverty, earning $1.9 or less per day. A 2020 report by the United Nations Development Program reveals that 1.3 billion people worldwide experience multidimensional poverty, lacking in areas such as health, education, and living standards. The ongoing pandemic and imminent economic recession have pushed more people into poverty, underscoring why the United Nations’ 2030 Sustainable Development Agenda prioritizes "ending poverty in all its forms everywhere" as its No. 1 sustainability goal. As engines of economic growth and national prosperity, businesses have a crucial role to play in global poverty eradication efforts [1]. Companies can contribute by providing products and services tailored to the needs of the bottom of the pyramid, donating funds and resources to impoverished communities, sourcing from underdeveloped regions, and offering employment opportunities to break the cycle of poverty [2]. Corporate poverty alleviation efforts are often viewed as acts of pure altruism, thus, it is worth exploring why profit-driven businesses would engage in supporting impoverished areas. The objective of this paper is to investigate the factors influencing corporate contributions to poverty alleviation.

We delve into this research topic within the context of China, a nation celebrated for its remarkable accomplishments in poverty reduction [3]. In 2016, the Chinese central government initiated the “Targeted Poverty Alleviation” campaign, aiming at lifting 7 million population above the poverty line by 2020 [4]. Throughout the process, the Chinese government enhanced the accountability of local village and county officials and ensured the active participation of key social entities in poverty eradication endeavors. Additionally, they implemented a range of measures aimed at combating poverty and fostering overall prosperity. These measures encompassed initiatives such as job creation, economic development in impoverished regions, relocation programs, educational and training programs, provisions for social welfare, investments in healthcare, and projects focused on environmental protection[5]. In line with the central government’s emphasis on poverty reduction, the China Securities Regulatory Commission (CSRC) announced its support for listed companies to actively participate in poverty alleviation efforts by implementing preferential measures for equity and debt financing applicants in impoverished regions, including firm’s initial public offerings (IPOs), bond issuances, and merger and acquisition (M&A) applications. Subsequently, both the Shenzhen and Shanghai Stock exchanges issued a notice to require listed firms to disclose corporate poverty alleviation efforts in their annual reports or standalone CSR reports starting from 2017.

These joint societal efforts to combat poverty have been highly successful. The China government then announced at the end of 2020 that all residents in China have been relieved from extreme poverty. In this process, Chinese listed firms play an important role. Based on our calculation (the data presented in this paragraph are derived from the enterprise poverty alleviation dataset within the CSMAR database), more than 40% of listed firms participated in the campaign between 2016 and 2019. These firms invested 260 billion Renminbi in poverty alleviation projects and helped 23.67 million deprived people out of poverty. One crucial feature of this national campaign is to involve all members of society, including listed firms, to contribute to nationwide poverty reduction endeavors. This provides us a great opportunity to empirically examine factors influencing corporate contribution to this crucial sustainable development goal.

Corporate contribution to poverty alleviation is often considered as a component of corporate philanthropy and corporate social responsibility (CSR) [6]. The institutional theory and stakeholder theory illuminate that CSR strategies frequently evolve in response to intricate social pressures, involving influences from government officials, local communities, industry peers, and others [7–10]. Thus, a firm’s determination to contribute to poverty reduction and the amount of such contributions are largely contingent on external factors it encounters. On one hand, empirical research has provided evidence supporting Johnson (1966)’s hypotheses regarding similar giving patterns within industries in philanthropic endeavors [11,12]. On the other hand, corporate philanthropy donations also manifest inter-regional disparities [13,14], because of regional governmental pressure [15,16] and resource endowments [17] on a firm’s inclination towards charitable giving and poverty reduction.

However, it is important to note that corporate contribution to poverty alleviation should not be completely conflated with corporate philanthropy. While CSR covers environmental, social, governance aspects [18], spanning economic, legal, ethical, and charitable responsibilities [19], corporate poverty alleviation targets poverty exclusively [4]. Moreover, corporate philanthropy involves voluntary, unconditional funds or asset transfers [20]. In contrast, corporate poverty alleviation extends beyond cash/material donations. It often collaborates with government or NGOs, creating jobs, engaging in diverse poverty reduction initiatives in disadvantaged regions. Given nuanced differences in scope, objectives, engagement, and intentions, prior studies on CSR predictors [21,22] and philanthropy antecedents [20,23] might not seamlessly apply to corporate poverty alleviation. This research, therefore, seeks to investigate whether China’s corporate contribution to poverty alleviation is affected by external factors at the industrial and provincial levels.

The study makes several contributions to the literature. First, we extend the empirical literature on CSR and charitable giving that has examined external determinants of firm contribution by the multilevel model (HLM) instead of the traditional OLS regression method. Traditional methods may lead to underestimating standard errors and violate the basic assumptions of OLS regression in terms of independence between observations, causing heteroscedasticity [24]. Therefore, we apply the multilevel model by nesting firm observations in industry group and province group and estimate more precisely and reliably the influence that multilevel factors have. Second, empirical studies have typically examined corporate philanthropy and corporate contribution to poverty reduction by investigating how industry competition, government intervention, and poverty rate shape firm behaviors [25–27]. From there, our studies conduct further analysis on more industry and region characteristics, such as a region’s economic prosperity, industry dynamism, and proportion of SOEs in industry, thus expanding the study on the determinants of corporate poverty alleviation and providing a comprehensive understanding of firm behaviors.

The remainder of this paper runs as follows. Section 2 presents the literature review. Section 3 develops our hypotheses. Section 4 offers the data used and the research design. Section 5 and section 6 reports the empirical and the additional analyses, respectively. Section 7 concludes the paper, presents implications, and proposes directions for future research.

## Literature review

Prior studies have generally examined corporate contributions to poverty reduction under the broader concept of corporate social responsibility (CSR) or corporate philanthropy [4,6,25]. Using the CSR data, it is commonly observed that firm size has garnered significant attention from scholars as a pivotal factor influencing CSR and philanthropic endeavors [12,28,29]. Larger firm size often correlates with heightened charitable activities. Furthermore, companies tend to display a greater propensity for engaging in socially responsible conduct when they possess surplus resources [30,31]. Additionally, other firm internal characteristics, such as social capital [32], ownership structure [12,33,34], and foreign direct investment [35], have been recognized as key determinants of CSR practices.

Beyond a firm’s inherent characteristics, some scholars argue that CSR practices are predominantly shaped by external social influences, irrespective of the immediate firm-level gains or cost, given firms’ intricate network of relationships and their corporate social behaviour are embedded in local societies [11,36–39]. From this perspective, the economic sector and institutional context in which a firm operates have been acknowledged as additional factors influencing its involvement in poverty reduction endeavors. First, Johnson’s (1966) groundbreaking study on corporate philanthropy concerning market structure revealed that industries characterized by either intense competition or significant monopolization do not create conducive environments for substantial charitable giving [40]. Further studies have also furnished evidence suggesting disparities in the inclination towards poverty alleviation across diverse sectors and industries [41,42]. Second, some researches have indicated that a peer effect plays a role in shaping charitable actions, poverty alleviation efforts, and other CSR practices, driven by normative and institutional pressures [26,42–47]. This implies that companies are inclined to engage in CSR initiatives when fellow companies within the same industry or community exhibit CSR behaviors. Moreover, other studies have highlighted that a firm’s CSR behaviors and strategies are also significantly influenced by factors such as political environment [48,49], social norms and values [50], government intervention [27,43], and other environment factors.

Among these studies, they employ traditional methods of analysis like OLS regression. Firm characteristics and their philanthropy and other CSR behaviors are all part of firm-level data. However, external social influences extend beyond the firm level, making traditional OLS regression inadequate for accommodating the hierarchical data structure. Hierarchical Linear Modeling (HLM) enables researchers to examine hierarchical data in a single comprehensive model and allows the measurement of variables and variances at different organizational levels [51]. It is therefore powerful for analyzing hierarchical data in which firm-level observations are clustered into higher-level organizations like industry and province. Thus, our research applies HLM to truly understand how industrial and provincial factors shape a firm’s poverty alleviation behavior.

## Hypotheses development

### Industry-level differences and corporate poverty alleviation

Studies on industry-level have increased in numbers in recent years, yet the relationship between industry competition and corporate contributions is controversial. One view points out that corporate philanthropy is more intense in oligopolistic, imperfectly competitive industries than in both monopolistic and highly competitive ones [40]. This is because firms often treat strategic, social responsibility behavior as a way to obtain a competitive advantage [20]. Monopolists do not need such a competitive advantage, while smaller firms in highly competitive sectors usually cannot afford charitable social causes [41]. In other words, industry competition and corporate contribution do not simply have a positive linear correlation. Another view argues that firms in competitive industries are more socially responsible and more generous in their charitable giving [52]. Similarly, the influence of industry competition to a poverty alleviation strategy also presents a mixed result.

Corporate poverty reduction projects and activities can help governments achieve their objectives and share social responsibilities, forming an approach for firms to obtain a competitive advantage and to gain access to scarce resources controlled by the government. With increasingly drastic market competition, more companies in China treat poverty alleviation as a tool and strategy to get access to the government’s various policy preferences and targeted project support and to obtain competitive advantages. It also cannot be ignored that, like other types of corporate philanthropy, contributions to poverty alleviation impose costs upon firms [53]. Firms operating under the efficiency principle, however, may prefer spending limited resources on core activities such as R&D and marketing instead of on corporate giving [54], especially for those that suffer severe competition when aiming to seize market share.

CSR behavior actually is a process that diverts shareholders’ money to social causes, but selfish investors in more competitive markets always pursue market returns, which result in the agency problem and hence a lower CSR contribution [52]. By the same token, intense competition may reduce the willingness and amount of a firm’s poverty reduction efforts. Consequently, for firms in a competitive field, whether they positively participate in poverty alleviation is unpredictable. Therefore, we expect that industry competition may not be a significant factor in shaping a corporate’s poverty alleviation contribution decision, and so we make the following predictions.

*Hypothesis 1a*. *Industry competition does not affect a firm’s preference to contribute to poverty alleviation activities*.
*Hypothesis 1b*. *Industry competition does not affect a firm’s preference to contribute substantially to poverty alleviation activities*.
*Hypothesis 1c*. *Industry competition does not affect a firm’s amount of investment in poverty alleviation activities*.

As a result of business-cycle fluctuations and potential changes, unpredictability, and volatility in regulatory contexts and industry features, firms are susceptible to high levels of uncertainty [55]. Such uncertainty impacts a firm’s managerial decisions regarding manufacturing and business strategies and consequences of its corporate behaviors [56–58]. Industry dynamism is often represented by the external uncertainty faced by firms [59–61]. Dynamism and uncertainty that firms confront include climate changes, natural disasters, changes in market demand and government regulations, as well as technology innovation [62,63]. Traditional research demonstrates that environmental uncertainty positively relates to a firm’s environment responsibility [64] and philanthropy donations [65], because participating in CSR enables it to obtain legitimacy [66,67] and to cope with risks that result from dynamism and fluctuation in the industry context [68,69]. However, dynamism increases the challenges of a firm’s resource allocation [70]. It further drives firms to adjust the allocation of limited existing resources in order to attract new sources of profit and to deal with their environmental dynamism [71].

The extent empirical research presents that a firm’s economic policy uncertainty negatively relates to philanthropic donations [72], while it positively relates to cash holdings [73]. Therefore, we expect that if firms compete in a more uncertain and dynamic industry, then they will pay more attention to their own business operations instead of participating in public welfare undertakings, such as poverty alleviation and philanthropic giving, that require human, material, and financial resources. This leads to the following hypotheses.

*Hypothesis 2a*. *For more dynamic industries*, *firms are less likely to contribute to poverty alleviation activities*.
*Hypothesis 2b*. *For more dynamic industries*, *firms are less likely to contribute substantially to poverty alleviation activities*.
*Hypothesis 2c*. *For more dynamic industries*, *firms will contribute less for poverty alleviation*.

Organizations’ operation activities are partly shaped by governmental institutions [74]. Among all the companies, SOEs have greater government interference [28,33] and less managerial discretion in corporate philanthropic giving [75]. Incompatible with traditional charitable business and to achieve the ambitious goal of eradicating poverty (i.e., lifting 98.99 million rural poor people and 28,000 poor villages out of poverty) in upcoming years, government departments taking on more pressure, and all firms and individuals are vital subjects who can help them meet the expected targets and results. Thus, SOEs are expected to have larger expenditures and undertake more responsibilities to help the government achieve the poverty eradication goal. Furthermore, firms pay close attention to what their peers are doing with a learning interaction process [76] and imitate them to develop various strategies, including CSR practices [44,45,77], which allow firms to obtain organizational legitimacy and public expectation [78].

Enterprises in the same industry often have similar endowments and face the same industrial policies and norms. Thus, the peer effect is more obvious within the industry [88], and firms tend to anchor the industry average [43]. More importantly, guided by State-owned Assets Supervision and Administration Commission (SASAC) of the State Council, which is the regulatory body of SOEs, expects them to be leading examples for all Chinese companies in social responsibility [47]. Combined, we expect that when the number of SOEs in an industry is larger, the industry demonstration effect is more likely to be generated so that other firms without poverty alleviation plans may be stimulated and follow SOEs to participate in poverty alleviation campaigns. Therefore, we propose the following hypotheses.

*Hypothesis 3a*. *In an industry with more SOEs*, *firms are more likely to contribute to poverty alleviation activities*.
*Hypothesis 3b*. *In an industry with more SOEs*, *firms are more likely to contribute substantially to poverty alleviation activities*.
*Hypothesis 3c*. *In an industry with more SOEs*, *firms will contribute more for poverty alleviation*.

### Provincial-level differences and corporate poverty alleviation

There is empirical evidence on CSR for poverty alleviation, emphasizing that firms in one or several regions show more pro-poor behaviors [25], because regions’ economic climate, institutional framework, culture, legal, government, and political differences, and utilization of production factors can seriously affect firm strategy [79,80]. Although the market is increasingly important in allocating resources in China, government organizations still maintain strong control of many crucial aspects of the economy [16]. Their influence on business operations is reflected in their allocation of scarce resources and in the design of industrial policies that grant protection to certain industries and sectors [81]. Combatting poverty and achieving collective prosperity in the nation are strategic deployments by the central committee.

China’s political system is characterized by a regionally decentralized authoritarian regime [82,83]. The governance structure describes the concept of a region, including central, provincial, city/county, and township governments that hold considerable power on allocating resources, approving new projects, and administration [84,85]. During the period of the “Targeted Poverty Alleviation” campaign, because of accountability of village and county officials, to fulfill the strategic goal of eradicating poverty in a short time, and to reduce governmental burdens, local authorities will transfer poverty alleviation pressures imposed by superior governments to local firms, and hence firms are expected and required to undertake more responsibilities in poverty reduction. Local government intervention varies greatly with the regions and provinces in China [86]. Local governments that pursue immediate regional economic performance may heavily intervene and distort firm decisions by exhibiting short-termism [87], and firms will be forced to rechannel their focus on social or political projects under such heavy intervention [88,89]. Therefore, in a province with strong government intervention, enterprises have a stronger motivation in poverty alleviation for obtaining more preferential policies and government subsidies. This leads to the following hypotheses.

*Hypothesis 4a*. *Firms headquartered in a province with greater government intervention are more likely to contribute to poverty alleviation activities*.
*Hypothesis 4b*. *Firms headquartered in a province with greater government intervention are more likely to contribute substantially to poverty alleviation activities*.
*Hypothesis 4c*. *Firms headquartered in a province with greater government intervention are associated a more investment in poverty alleviation activities*.

The traditional view suggests that an unhealthy economy will constrain CSR being embedded in firms [10]. Companies are less likely to apply social responsibility if they operate in a climate where inflation is high, productivity growth is low, consumer confidence is weak, etc., because CSR’s contribution will make it relatively difficult to gain healthy profits in the near term under these conditions [90]. However, different from other charitable donations, China’s anti-poverty campaign that eliminates extreme poverty has different provincial conditions, as the poverty rate for individual localities may be higher or lower. Some provinces even have finished before 2020 (the expected year to achieve the goal). For instance, in 2013, Beijing, Shanghai, and Tianjin realized a poverty rate of 0, while 17 other provinces successfully reached the poverty alleviation goal by 2019. Obviously, the pressure and concentration on local governments for the “Targeted Poverty Alleviation” campaign increase as the number of poor people and the poverty rate under the jurisdiction rise.

Medina-Muñoz & Medina-Muñoz (2020) pointed out that the higher the poverty rate is in a region, the greater the importance of adopting pro-poor CSR initiatives should be [25]. Accordingly, if a company operates in a province with more people living in poverty, then to fulfill the task of alleviation poverty firms might be expected and required by governments to contribute more in favor of the poor. The government and relevant departments have power to evaluate the appropriateness of corporate behavior and to allocate scarce resources and favorable treatments, such as land, bank loans, subsidies, tax breaks, or government contracts [81]. To gain access to resources and obtain favored treatments, enterprises will strive to meet the government’s expectations and help local authorities achieve the goal of the anti-poverty campaign. Hence, we formulate the following hypotheses.

*Hypothesis 5a*. *Firms headquartered in a province with a higher poverty rate are more likely to contribute to poverty alleviation activities*.
*Hypothesis 5b*. *Firms headquartered in a province with a higher poverty rate are more likely to contribute substantially to poverty alleviation activities*.
*Hypothesis 5c*. *Firms headquartered in a province with a higher poverty rate are associated with more investment in poverty alleviation activities*.

Unbalanced regional development means that the prosperity of provinces varies. On one hand, for a prosperous province, local authorities have comparatively abundant financial resources to spare for assisting poor people and villages and for achieving the goal of eliminating extreme poverty with less local firms’ help, which can lead to corporates voluntarily undergoing poverty eradication. Considering the efficiency and cost-benefit, firms may spend limited organizational internal resources to more profitable and lucrative activities that are urgent and in need of by local governments so as to obtain exchangeable resources and acquirable benefits or to respond to institutional pressures exerted by the government. On the other hand, an increase in GDP is associated with reductions in poverty [91,92]. In other words, the prosperity of a province reflects a smaller number of poor people, which means that the province has a better economic climate with less need for helping the poor. In short, a firm’s willingness toward helping poverty alleviation negatively relates to local economic conditions. Thus, we develop the next hypotheses.

*Hypothesis 6a*. *Firms headquartered in a more prosperous province are less likely to contribute to poverty alleviation activities*.
*Hypothesis 6b*. *Firms headquartered in a more prosperous province are less likely to contribute substantially to poverty alleviation activities*.
*Hypothesis 6c*. *Firms headquartered in a more prosperous province are associated with less investment in poverty alleviation activities*.

## Data, variables, and methodology

### Variable measurements

We test the influence of industry factors and provincial factors on a firm’s poverty alleviation willingness and contribution. The dependent variables are *Poverty activity*, *Poverty investment*, and *Investment amount*. *Poverty activity* is an indicator variable to capture whether a firm participates in the anti-poverty drive; it equals 1 when a firm has reported that it is involved in poverty alleviation in its corporate annual reports and 0 otherwise. *Poverty investment* is another indicator variable to capture whether a firm makes a substantial contribution to the anti-poverty drive; it equals 1 when a firm invests money and/or materials in poverty alleviation and 0 otherwise. *Investment amount* is a continuous variable to measure the amount of poverty alleviation contribution, which is measured as the natural log of the sum of a firm’s contribution in monetary and material discounts to the anti-poverty drive. We set the value of *Investment amount* to zero for firms with no record of poverty alleviation commitment in corporate annual reports or have no substantial investment in the anti-poverty drive—that is, *Investment amount* equals 0 if *Poverty activity* is 0 or *Poverty investment* is 0.

The independent variable are various industrial and provincial factors that are related to the hypotheses. Industrial factors are *HHI*, Dynamism, and *SOE ratio*. *HHI* (i.e., Herfindahl-Hirschman Index) is a measure for industry competition intensity, calculated by squaring the sales revenue of each firm competing in the industry and then summing the resulting numbers. A higher *HHI* represents a greater degree of monopoly and lower competition. Industry is based on the classification provided by China Securities Regulatory Commission (CSRC). We follow Ghosh & Olsen (2009) to measure *Dynamism*, using the variation of sales to capture the change and volatility of the industrial environment [70]. Specifically, we calculate the variance of industry three-year rolling sales revenue growth rate as industry dynamism, where a higher *Dynamism* means greater volatility. The *SOE ratio* is measured as the number of SOEs by the total number of firms in the industry. We expect *HHI* to not have a significant coefficient, while *Dynamism* has a negative coefficient, and the *SOE ratio* has a positive coefficient, which support H1 to H3.

Provincial factors are *Gov_Intervention*, *Poverty rate*, and *GDP per capita*. *Gov_Intervention* is measured as the level of provincial government intervention using the Government and Market Relationship (GMR) index of the province in which a firm is headquartered [53,86]. According to Wang et al. (2018), the GMR index is constructed using three factors: (1) the ratio of provincial government revenue to its GDP; (2) the ratio of average time spent by a firm’s manager in dealing with the government to weekly average working hours; and (3) the ratio of government employees to provincial population [93]. A higher GMR index captures a lower degree of government intervention. Just to make the results more intuitive and easier to understand, we measure *Gov_Intervention* as the opposite value of the GMR index, which represents that a higher *Gov_Intervention* indicates greater government intervention. *Poverty rate* indicates the provincial incidences of poverty, measured as the number of people living below the poverty line by a province’s total population. *GDP per capita* captures provincial prosperity. We expect *Gov_intervention* to have a positive coefficient, *Poverty rate* to have a positive coefficient, and *GDP per capita* to have a negative coefficient, which support H4 to H6.

Following prior research [4,94], we control a set of variables. First, we include six firm characteristics. *Size* is measured as the natural log of sales revenue in the year. *Age* is measured as the number of years since the firm’s establishment. *Leverage* is measured as total liabilities divided by total assets in the year. *ROA* is measured as net income divided by total assets in the year. *Book-to-market* is calculated by shareholders’ equity divided by market value in the year. *Growth* is calculated by annual sales revenue growth rate. Second, we incorporate governance variables and executive characteristics variables as controls. *SOE* (i.e., state ownership) is a dummy variable that equals 1 when the firm is a state-owned enterprise and 0 otherwise. *Dual* is a dummy variable, equaling 1 if a firm’s entrepreneur serves as the CEO and the chairman simultaneously and 0 otherwise. *Independence* is the percentage of independent directors. *Board size* is measured as the natural log of the number of a firm’s board members. *Largest_SH* measures ownership concentration, calculated by the percentage of shares owned by the firm’s top one shareholder. *Ave_Compensation* is measured as the average natural log of total compensation of a firm’s top management team, and *Ave_Share* is measured as the average percentage of shares owned by the firm top management team. In addition, because industry and community peers influence corporate philanthropy [16], *Ind_Amount* (i.e., average poverty alleviation contribution of industry peers for each year, except for the focal firm) and *Prov_Amount* (i.e., average poverty alleviation contribution of province peers for each year, except for the focal firm) are incorporated as control variables. We summarize the measurements of these variables in Table 1.

**Table 1:** Variable measurements.

Variable	Description
**Dependent variables**	
Poverty activity	dummy variable that equals 1 when the firm reports poverty alleviation in the corporate annual reports and 0 otherwise
Poverty investment	dummy variable that equals 1 when the firm inputs fund or materials into anti-poverty drive and 0 otherwise
Investment amount	natural log of the sum of a firm’s monetary and material discounts’ contribution to poverty alleviation
**Independent variables**	
HHI^1^	Herfindah-Hirschman Index for each industry
Dynamism	variance of industry three-year rolling sales revenue growth rate
SOE ratio	number of state-owned firms in an industry to total firms in the industry
Gov_Intervention	government and market relationship (GMR) index of the province (take the opposite number)
Poverty rate^2^	number of people living below the poverty line within province’s total population
GDP per capita	provincial per capita gross domestic product (GDP)
**Control variables**	
Size	natural log of total assets
Age	natural log of number of years since the firm’s establishment
Leverage	total debt divided by total assets
ROA	net income divided by total assets
Book-to-market	shareholders’ equity divided by market value
Growth	annual sales revenue growth rate
SOE	state ownership; a dummy variable that equals 1 when the firm is a state-owned enterprise and 0 otherwise
Dual	CEO duality; a dummy variable that equals 1 when one person occupies both board chair and CEO positions and 0 otherwise
Independence	percentage of independent directors
Board size	natural log of the number of a firm’s board members
Largest_SH	percentage of shares owned by the firm’s top one shareholders
Ave_Compensation	average of the natural log of total compensation of TMT
Ave_Share	average of the percentage of shares owned by a firm’s TMT
Ind_Amount^3^	average poverty alleviation contribution of industry peers for each year, except for the focal firm
Prov_Amount^4^	average poverty alleviation contribution of province peers for each year, except for the focal firm

^1^ HHI = sum [(X_i_ / X)^2^], where X is the sales revenue of firm *i* in the industry, and *X* is the total sales revenue for all firms in the industry. A lower value of HHI indicates higher competition.

^2^ Data are compiled by Department of Household Surveys National Bureau of Statistics of China, and sources come from Poverty Monitoring Report of Rural China (2020).

^3^ We define industry peers as firms with the same two-digit industrial classification code as the focal firm.

^4^ We define province peers as firms headquartered in the same province as the focal firm.

### Data and sample identification

Our sample includes Chinese firms listed in Shanghai and Shenzhen Stock Exchanges between 2016 and 2019. Our sampling period starts in 2016, the first year when Chinese listed firms are required to disclose their poverty alleviation activities in their annual reports or standalone corporate social responsibility reports. To enhance the robustness of results, we exclude financial firms (with CSRC industry code J) and special treatment (ST) companies within the study period. After excluding observations with missing data, we have a final sample of 8415 firm-year observations from 2016 to 2019. We source the data of GDP per capita at the province-level from the China Statistical Yearbook, provincial poverty rate from Poverty Monitoring Report of Rural China (2020), and government intervention from Marketization Index of China’s Provinces [93]. The firm poverty alleviation contribution and financial statement data are obtained from the China Stock Markets and Accounting Research Database (CSMAR).

### Methodology: Multilevel model

Our research tests the association between industrial and provincial factors with a firm’s willingness and contribution toward the anti-poverty drive. In the study, firm poverty alleviation data have a hierarchical nature—that is, firm observations are nested in industry group and province group, and the data have different levels of aggregation. In addition to their hierarchical nature, the data have a longitudinal nature—industrial and provincial characteristic variables are repetitive observations and measurements for the same firm over the years. Therefore, the assumption of independence of errors may be violated when using traditional single-level methods [95]. A multilevel model is able to examine hierarchical data in a single comprehensive model and allows the measurement of variables and variances at different organizational levels [51]. This method is appropriate for examining how effects at a given level are shaped by factors nested at different levels [96,97]. It is widely used in social sciences, medicine, healthcare, and economics literature; however, it is relatively new to accounting [98]. Based on the aim of this research, we conduct two 2-level models to examine the effect of industry characteristics on a firm’s poverty alleviation with firms (level-1) that are nested within industry (level-2) and province characteristics on a firm’s poverty alleviation with firms (level-1) that are nested within a province (level-2).

The first model is the Null model, which allows the mean for group (industry or province) *j* to depart randomly from the overall mean of firm poverty alleviation (dependent variables *Y*_*ij*_) by an amount *μ*_0*j*_. We can determine how much of the variance in firm poverty alleviation lies between industries of provinces. The Null model for firm *i* in group *j* is represented as follows:

$$\begin{array}{l} Level‐1:\;Y_{ij}=\beta_{0j}+r_{ij}\\ Level‐2:\;\beta_{0j}=\gamma_{00}+\mu_{0j} \end{array}$$ (1)

Level-1 (firm-level) equation corresponds to the poverty alleviation of each firm as a function of an industry or province mean and random error. The dependent variable *Y*_*ij*_ is the willingness and contribution on poverty alleviation of firm i in group *j*. The coefficient *β*_0*j*_ is the fixed effect of level-1 intercept, which represents the average firm poverty alleviation of group *j*, and *r*_*ij*_ is a random firm effect (the deviation of firm *ij*’s score from the industry / province mean).

Level-2 (industry-level or province-level in terms of different research purpose) equation corresponds to the variability among industries or provinces. Here, *β*_0*j*_ is simultaneously modeled as an outcome varying randomly around a group mean. In level-2 equation, the level-1 equation coefficient *β*_0*j*_ is used as an outcome variable related to each of the level-2 predictors. The random group effect *μ*_0*j*_ is the error, which represents the group differences. The variance of *μ*_0*j*_, donated by τ_00_, quantifies the degree of heterogeneity in intercepts across groups, and the variance of *γ*_*ij*_, denoted by *σ*^2^, quantifies the within-group variance.

As part of the Null model building process, it is necessary to use intraclass correlation (ICC) to quantify the proportion of total variation in firm poverty alleviation accounted for by group differences. ICC = τ_00_/(τ_00_+*σ*^2^). A value near zero suggests that only a model including level-1 variables is appropriate, and there is no need to use a multilevel model. Aguinis et al. (2013) conducted a literature review and found that the ICC value reported usually ranges from 0.10 to 0.30, while some reported a smaller ICC value from 0.05 to 0.20 [97].

The second model is the Full model. Level-1 is the firm-level, while level-2 is the industry-level or province-level in our research. The industry-level predictive model explains how differences in industry variables may influence a firm’s poverty alleviation strategy within each industry, and the province-level predictive model explains how differences in province variables may influence a firm’s poverty alleviation strategy within each province. The model involves firm-level control variables in level-1 and industry-level (or province-level) independent variables in level-2 as shown by the following:

$$\begin{array}{c} Level‐1:\;Y_{ij}=\beta_{0j}+\beta_{1}{controls}_{1ij}+\cdots +\beta_{n}{controls}_{nij}+r_{ij}\\ {Level‐2:\;\beta }_{0j}=\gamma_{00}+\gamma_{01}X_{1j}+\cdots +\gamma_{0n}X_{nj}+\mu_{0j} \end{array}$$ (2)

Here, *β*_*n*_ are the fixed slopes that represent the average effect of the control variables (including *Size*, *Age*, *Leverage*, *ROA*, *Book-to-market*, *Growth*, *SOE*, *Dual*, *Independence*, *Board size*, *Largest_SH*, *Ave_Compensation*, *Ave_Share*, *Ind_Amount*, and *Prov_Amount*) on the poverty alleviation willingness and contribution across the sample of firms. The random firm effect *r*_*ij*_ represents residual variance not explained by these firm-level independent variables added to the Null model. *γ*_0*n*_ is the regression coefficients for the group-level independent variables. When we examine the influence of industry factors, *X*_*nj*_ represent *HHI*, *Dynamism*, and *SOE ratio*, respectively. When we examine the influence of province factors, *X*_*nj*_ represent *Gov_Intervention*, *Poverty rate*, and *GDP per capita*, respectively.

## Multivariate analysis results

### Descriptive statistics and variable correlations

Table 2 presents descriptive statistics for key variables used in the multilevel models. On average, only 31.8% of firms participated in the poverty alleviation campaign, and 84.5% of them invested money and/or materials, while about 15.5% of firms did not make any substantial contribution. The average amount of poverty alleviation investment is 7.871 million Renminbi, and the maximum investment is 8.669 billion Renminbi. It is noted that 68.2% of the samples’ *Investment amount* is zero, and so that does not obey a standard normal distribution. Thus, according to the literature [99,100], we adopt the Tobit regression approach instead of OLS regression to test the hypotheses when the independent variable is *Investment amount*.

**Table 2:** Descriptive statistics.

Variable	N	mean	sd	min	p50	max
Poverty activity	8,415	0.318	0.466	0.000	0.000	1.000
Poverty investment	2,674	0.845	0.362	0.000	1.000	1.000
Investment amount (Renminbi mn)	8,415	7.871	154.141	0.000	0.000	8669.970
HHI	8,415	0.135	0.140	0.028	0.095	1.000
Dynamism	8,415	0.042	0.100	0.001	0.027	2.000
SOE ratio	8,415	0.305	0.206	0.000	0.233	1.000
Gov_Intervention	8,415	-6.734	2.454	-8.830	-6.890	26.240
Poverty rate	8,415	1.377	2.395	0.000	0.000	13.200
GDP per capita	8,415	11.238	0.391	10.227	11.301	11.851
Size (log)	8,415	22.253	1.306	17.779	22.111	28.509
Age	8,415	19.288	5.442	4.000	19.000	52.000
Leverage	8,415	0.416	0.208	0.010	0.404	3.919
ROA	8,415	0.034	0.113	-4.946	0.038	0.384
Book-to-market	8,415	0.345	0.155	-0.333	0.329	1.290
Growth	8,415	0.726	7.733	-11.924	0.166	434.593
Largest_SH	8,415	33.908	14.299	4.078	31.845	88.235
SOE	8,415	0.317	0.465	0.000	0.000	1.000
Dual	8,415	0.298	0.457	0.000	0.000	1.000
Independence	8,415	0.376	0.055	0.200	0.364	0.800
Board size	8,415	8.415	1.665	3.000	9.000	18.000
Ave_Compensation (log)	8,415	13.512	0.711	0.000	13.499	16.521
Ave_Share	8,415	0.027	0.041	0.000	0.003	0.594
Ind_Amount (log)	8,415	1.012	0.739	0.000	0.807	8.023
Prov_Amount (log)	8,415	1.016	0.610	0.084	0.851	4.869

Table 3 reports the correlation coefficients among the key variables. We find that *SOE ratio*, *Gov_Intervention*, and *Poverty rate* are positively associated with three dependent variables, *Dynamism* and *GDP per capita* are significantly and negatively associated with all the dependent variables, while *HHI* is unrelated to poverty activity and investment. These findings provide preliminary support to all the hypotheses. In other words, a firm’s willingness and contribution on poverty alleviation relate to industrial and provincial factors.

**Table 3:** Correlation matrix of key variables.

	**Variable**	**1**	**2**	**3**	**4**	**5**	**6**	**7**	**8**
1	Poverty activity	1.000							
2	Poverty investment	-	1.000						
3	Investment amount	0.784*	0.656*	1.000					
4	HHI	0.014	-0.022	0.023*	1.000				
5	Dynamism	-0.021*	-0.037	-0.027*	0.236*	1.000			
6	SOE ratio	0.214*	0.089*	0.223*	0.178*	0.007	1.000		
7	Gov_Intervention	0.192*	0.081*	0.173*	0.035*	0.011	0.118*	1.000	
8	Poverty rate	0.218*	0.073*	0.181*	0.054*	-0.013	0.185*	0.552*	1.000
9	GDP per capita	-0.192*	-0.090*	-0.157*	-0.005	0.022*	-0.162*	-0.413*	-0.778*
10	Size	0.288*	0.083*	0.381*	0.088*	-0.027*	0.344*	0.037*	0.037*
11	Age	0.099*	0.045*	0.081*	0.030*	0.024*	0.168*	0.001	0.038*
12	Leverage	0.119*	0.022	0.153*	0.065*	-0.005	0.217*	0.027*	0.073*
13	ROA	0.045*	0.021	0.055*	-0.077*	-0.019	-0.013	-0.005	-0.023*
14	Book-to-market	0.017	0.042*	0.015	0.003	0.009	0.023*	-0.015	-0.124*
15	Growth	-0.012	0.008	-0.011	0.016	-0.004	0.033*	0.008	0.022*
16	Largest_SH	0.096*	0.040*	0.116*	0.059*	-0.023*	0.200*	0.024*	0.011
17	SOE	0.244*	0.123*	0.227*	0.072*	0.006	0.450*	0.100*	0.175*
18	Dual	-0.089*	-0.064*	-0.090*	-0.056*	-0.006	-0.172*	-0.068*	-0.115*
19	Independence	-0.025*	0.026	0.003	0.003	0.001	-0.065*	0.021	-0.008
20	Board size	0.135*	0.047*	0.142*	0.034*	-0.007	0.199*	0.030*	0.078*
21	Ave_Compensation	0.075*	-0.016	0.115*	-0.008	0.014	0.001	-0.082*	-0.166*
22	Ave_Share	-0.173*	-0.095*	-0.171*	-0.074*	-0.008	-0.281*	-0.112*	-0.153*
23	Ind_Amount	0.244*	0.085*	0.260*	0.121*	-0.031*	0.618*	0.182*	0.144*
24	Prov_Amount	0.257*	0.091*	0.229*	0.002	0.037*	0.113*	0.615*	0.529*
	**Variable**	**9**	**10**	**11**	**12**	**13**	**14**	**15**	**16**
9	GDP per capita	1.000							
10	Size	-0.022*	1.000						
11	Age	-0.069*	0.151*	1.000					
12	Leverage	-0.067*	0.513*	0.169*	1.000				
13	ROA	0.021	0.028*	-0.038*	-0.337*	1.000			
14	Book-to-market	0.099*	0.037*	-0.025*	-0.477*	0.132*	1.000		
15	Growth	-0.023*	0.018	0.016	0.021	0.005	-0.016	1.000	
16	Largest_SH	0.020	0.175*	-0.044*	0.042*	0.104*	0.038*	-0.008	1.000
17	SOE	-0.120*	0.357*	0.249*	0.250*	-0.018	-0.037*	0.003	0.203*
18	Dual	0.105*	-0.187*	-0.133*	-0.122*	0.019	0.011	-0.006	-0.025*
19	Independence	0.031*	-0.026*	-0.029*	-0.018	-0.017	-0.026*	0.009	0.048*
20	Board size	-0.076*	0.263*	0.108*	0.137*	0.017	0.019	-0.006	0.006
21	Ave_Compensation	0.154*	0.332*	0.056*	0.102*	0.084*	0.023*	-0.055*	-0.002
22	Ave_Share	0.140*	-0.360*	-0.250*	-0.271*	0.097*	0.126*	-0.021	-0.057*
23	Ind_Amount	-0.162*	0.255*	0.165*	0.127*	0.018	0.112*	0.003	0.146*
24	Prov_Amount	-0.509*	0.033*	0.088*	0.056*	-0.033*	0.070*	-0.003	-0.002
	**Variable**	**17**	**18**	**19**	**20**	**21**	**22**	**23**	**24**
17	SOE	1.000							
18	Dual	-0.292*	1.000						
19	Independence	-0.061*	0.123*	1.000					
20	Board size	0.260*	-0.187*	-0.574*	1.000				
21	Ave_Compensation	0.003	-0.069*	-0.029*	0.099*	1.000			
22	Ave_Share	-0.423*	0.227*	0.087*	-0.204*	-0.004	1.000		
23	Ind_Amount	0.266*	-0.109*	-0.027*	0.138*	0.012	-0.187*	1.000	
24	Prov_Amount	0.143*	-0.072*	0.033*	0.035*	-0.034*	-0.086*	0.267*	1.000

* indicates that the results are significant at the 5% level.

The VIF test shows that the value of VIF is between 1.01 to 3.18, and the mean VIF is 1.70.

### Null model and ICC test

Table 4 presents the ICC test of the null model. Considering the industrial effect on a firm’s poverty alleviation, the ICC values in the three regressions are 0.208, 0.095, and 0.184, respectively. Results suggest that 20.8% of the total variation in the likelihood of a firm’s poverty alleviation lies between industries, 9.5% of the total variation in the likelihood of a firm’s willingness to invest funds or materials into poverty alleviation lies between industries, and 18.4% of the total variation in the amount of a firm’s contribution on poverty alleviation lies between industries. Similarly, after considering the industrial effect on a firm’s poverty alleviation, the ICC values in the three regressions are 0.180, 0.090, and 0.126, respectively, meaning that over 9% of the total variation in a firm’s poverty contribution can be explained by the province. In conclusion, the variation of the study’s variables is decomposed into level-1 and level-2, and thus it is appropriate to apply the multilevel model to do the research.

**Table 4:** Results of the null model and ICC test.

Group	Industry			Province		
Dependent variable	(1) Probit	(2) Probit	(3) Tobit	(4) Probit	(5) Probit	(6) Tobit
	Poverty activity	Poverty investment	Investment amount	Poverty activity	Poverty investment	Investment amount
Intercept	-0.443***	1.053***	-3.364***	-0.201**	1.159***	-2.204***
	(0.066)	(0.058)	(0.388)	(0.087)	(0.074)	(0.451)
Variance[_cons]	0.262***	0.104***	8.321***	0.220***	0.099**	5.619***
	(0.056)	(0.037)	(1.785)	(0.062)	(0.046)	(1.557)
ICC	0.208	0.095	0.184	0.180	0.090	0.126
Observations	8,415	8,415	8,415	8,415	8,415	8,415
Number of groups	72	72	72	31	31	31
Year	YES	YES	YES	YES	YES	YES

When dependent variables are *Poverty activity* and *Poverty investment* (dummy variable), we use the Probit model for the test. When dependent variable is *Investment amount* (68.2% samples have a zero value), we use the Tobit model for the test.

*** p<0.01

** p<0.05, and

* p<0.1.

Robust standard errors are reported in parentheses.

### Multivariable analysis

Table 5 shows the results of multilevel regressions, with column 1–3 testing the association between industrial factors (i.e., *HHI*, *Dynamism*, and *SOE ratio*) and a firm’s contribution on poverty alleviation, and column 4–6 testing the association between provincial factors (i.e., *Gov_Intervention*, *Poverty rate*, and *GDP per capita*) and a firm’s contribution on poverty alleviation. The table represents that *HHI* does not influence a firm’s strategy on participating in the anti-poverty campaign—that is, whether firms compete in a competitive industry or not, their willingness to help the poor will not be affected. Thus, H1a, H1b, and H1c are supported.

**Table 5:** Results of the full model.

Group	Industry			Province		
Variable	(1) Probit	(2) Probit	(3) Tobit	(4) Probit	(5) Probit	(6) Tobit
	Poverty activity	Poverty investment	Investment amount	Poverty activity	Poverty investment	Investment amount
Constant	-7.572***	-0.163	-43.890***	-2.802	6.514**	-13.354
	(0.499)	(0.946)	(2.510)	(2.546)	(2.729)	(12.737)
HHI	-0.089	-0.088	0.219			
	(0.231)	(0.302)	(1.251)			
Dynamism	-0.173	-0.450*	-1.912**			
	(0.185)	(0.256)	(0.960)			
SOE ratio	0.808***	0.517*	5.178***			
	(0.246)	(0.289)	(1.310)			
Gov_Intervention				0.063***	0.088**	0.212***
				(0.021)	(0.036)	(0.072)
Poverty rate				0.032	-0.034	0.151*
				(0.020)	(0.027)	(0.089)
GDP per capita				-0.429**	-0.631***	-2.917***
				(0.216)	(0.222)	(1.093)
Size	0.267***	0.053	1.560***	0.295***	0.094***	1.767***
	(0.019)	(0.037)	(0.093)	(0.019)	(0.036)	(0.091)
Age	0.101*	0.028	0.477*	0.116**	0.056	0.523*
	(0.053)	(0.106)	(0.274)	(0.053)	(0.105)	(0.272)
Leverage	-0.376***	0.008	-1.614**	-0.586***	-0.239	-3.060***
	(0.132)	(0.270)	(0.683)	(0.125)	(0.258)	(0.642)
ROA	0.473**	0.318	3.732***	0.579***	0.321	4.469***
	(0.190)	(0.322)	(1.079)	(0.189)	(0.314)	(1.081)
Book-to-market	-0.360**	0.362	-1.353*	-0.346**	0.170	-1.603**
	(0.144)	(0.292)	(0.731)	(0.139)	(0.281)	(0.699)
Growth	-0.002	0.003	-0.010	-0.004	0.004	-0.017
	(0.003)	(0.009)	(0.013)	(0.003)	(0.010)	(0.013)
Largest_SH	0.002	-0.001	0.006	0.003**	-0.000	0.011**
	(0.001)	(0.002)	(0.006)	(0.001)	(0.002)	(0.006)
SOE	0.320***	0.227***	1.425***	0.311***	0.253***	1.398***
	(0.040)	(0.080)	(0.205)	(0.039)	(0.079)	(0.199)
Dual	0.009	-0.043	0.004	0.051	-0.054	0.210
	(0.036)	(0.075)	(0.190)	(0.036)	(0.074)	(0.189)
Independence	0.254	1.540**	3.027*	-0.118	1.437**	1.432
	(0.348)	(0.707)	(1.723)	(0.350)	(0.699)	(1.718)
Board size	0.251**	0.226	1.334***	0.233**	0.170	1.254**
	(0.101)	(0.191)	(0.503)	(0.101)	(0.188)	(0.496)
Ave_Compensation	0.003	-0.106**	-0.025	0.009	-0.071	0.008
	(0.023)	(0.052)	(0.116)	(0.024)	(0.048)	(0.117)
Ave_Share	-0.822*	-1.269	-5.058**	-0.848*	-1.261	-5.450**
	(0.471)	(1.016)	(2.527)	(0.469)	(1.015)	(2.500)
Ind_Amount	-0.187***	-0.121	-1.780***			
	(0.073)	(0.084)	(0.349)			
Prov_Amount				-0.057	-0.214*	-0.657
				(0.108)	(0.126)	(0.504)
Observations	8,415	2,674	8,415	8,415	2,674	8,415
Number of groups	72	68	72	31	31	31
Year	YES	YES	YES	YES	YES	YES

When dependent variables are *Poverty activity* and *Poverty investment* (dummy variable), we use the Probit model for the test. When dependent variable is *Investment amount* (68.2% samples have a zero value), we use the Tobit model for the test.

All independent and control variables are lagged for one year.

*** p<0.01

** p<0.05 and

* p<0.1.

Robust standard errors are reported in parentheses.

As for the relationship between industrial variables and a firm’s poverty alleviation, the table demonstrates that the coefficient of *Dynamism* is negative but not significant in column 1. However, it is significantly positive in columns 2 (γ = 0.450, p<0.1) and 3 (γ = 1.912, p<0.05). This suggests that industry uncertainty will not affect firms’ willingness to participate in poverty alleviation, but is negatively associated with firms’ willingness to make monetary and material contribution. For more uncertain industries, firms will contribute less for poverty alleviation. The results therefore provide weak support to H2b and strong support to H2c, while H2a cannot be supported. This may be because firms have various ways to act in the anti-poverty campaign, except for substantial contribution (i.e., money). Companies can assist the poor by creating flyers, establishing educational and training programs to help increase employability of the poor, or donating their existing inventories, supplies, products, or other materials to poverty alleviation projects without spending less additional resources and affecting operational cash flow. Hence, whether firms face high uncertainty or not, their willingness to take part in poverty activities is hard to be influenced.

The table shows a statistically significant positive correlation between *SOE ratio* and a firm’s contribution on poverty alleviation in terms of *Poverty activity* (γ = 0.808, p<0.01), *Poverty investment* (γ = 0.517, p<0.1), and *Investment amount* (γ = 5.178, p<0.01). This means in an industry with more SOEs that firms have stronger willingness to participate in poverty alleviation, as well as make more substantial (money or materials) contributions. Empirical findings support H3a, H3b, and H3c, with H3b being relatively weakly supported.

As for the relationship between provincial variables and a firm’s poverty alleviation, we first observe a statistically significant relationship between *Gov_Intervention* and a firm’s contribution on the anti-poverty drive, no matter whether considering *Poverty activity* (γ = 0.063, p<0.01), *Poverty investment* (γ = 0.088, p<0.05), and *Investment amount* (γ = 0.212, p<0.01). The result indicates that firms increase initiative and positivity to participate in the poverty alleviation campaign as well as make more substantial monetary and material contributions when they are headquartered in a province with greater government intervention. This suggests that a poverty alleviation strategy is one method for firms to maintain relationships with governments. Hypotheses 4a, 4b, and 4c are thus supported.

We also find that the relationship between a province’s poverty incidence (i.e., poverty rate) and a firm’s intention for helping the poor and making a substantial contribution has no statistical significance. Moreover, a province’s *Poverty rate* positively relates to a firm’s *Investment amount* (including fund and materials) on poverty alleviation activities and is significant at the 10% level. This indicates that higher poverty incidences in a province denote a greater amount of poverty alleviation invested by firms. The outcome provides weakly support for H5c (at the 10% significance level), but does not support H5a and H5b.

We finally see that higher *GDP per capita* in a province is associated with a less likelihood of being involved in supporting the poor, as predicted by H6a, H6b, and H6c. The coefficient of *GDP per capita* is -0.429 (p<0.05), -0.631 (p<0.01), and 2.917 (p<0.01) in columns 4 to 6, respectively. The result means that if a firm is headquartered in a more prosperous province, then it will show less willingness to participate in poverty alleviation activities and make substantial contributions. Moreover, a more prosperous province will lead to a decrease in funds and material contribution to poverty alleviation.

## Additional analysis

In our main analysis we do not differentiate between cash contribution and material contribution, but instead use the aggregated amounts of these two types of contributions to test our hypotheses. However, executives’ willingness and firms’ ability to contribute may affect cash and material contributions in a distinct way. Cash contribution is drawn from a firm’s operating cash flow and imposes a direct cost to it. In contrast, material contribution can take a wide variety of forms; e.g., firms can choose to donate their existing inventories, supplies, products, or other materials to poverty alleviation projects. This action is typically much cheaper and does not affect operating cash flow. We next reexamine our main hypotheses by separating these two types of contributions with “Fund” indicating the amount of cash contribution and “Material” referring to the amount of material contribution.

Table 6 presents the results. Odd columns exhibit the Null model test, and all the ICC values are larger than 0.100. This means that over 10% of the total variation in a firm’s poverty contribution can be explained by industry and province, so that applying the multilevel model is appropriate. We see that firms competitive in an industry under a less dynamic environment are more likely to invest funds into poverty alleviation, while environment uncertainty is not associated with material contribution. This may because material donation does not influence a firm’s operating cash flow and imposes a direct cost to firms.

**Table 6:** Results of additional analysis.

Group	Industry				Province			
Variable	(1) Tobit	(2) Tobit	(3) Tobit	(4) Tobit	(5) Tobit	(6) Tobit	(7) Tobit	(8) Tobit
	Fund	Fund	Material	Material	Fund	Fund	Material	Material
Constant	-3.591***	-44.410***	-5.792***	-32.174***	-2.510***	-23.914***	-4.639***	-7.747
	(0.386)	(2.534)	(0.358)	(2.468)	(0.436)	(9.051)	(0.394)	(6.487)
HHI		0.517		-1.729				
		(1.256)		(1.093)				
Dynamism		-1.915**		-0.503				
		(0.965)		(0.922)				
SOE ratio		4.809***		2.345**				
		(1.309)		(0.990)				
Gov_Intervention						0.133**		0.106***
						(0.057)		(0.040)
Poverty rate						0.152*		0.120*
						(0.081)		(0.069)
GDP per capita						-1.929**		-2.345***
						(0.795)		(0.552)
Controls	YES	YES	YES	YES	YES	YES	YES	YES
ICC test	0.179		0.185		0.116		0.142	
Observations	8,415	8,415	8,415	8,415	8,415	8,415	8,415	8,415
Number of groups	72	72	72	72	31	31	31	31
Year	YES	YES	YES	YES	YES	YES	YES	YES

Multilevel Tobit model is applied in doing the additional test.

All independent and control variables are lagged for one year.

*** p<0.01

** p<0.05, and

* p<0.1. Robust standard errors are reported in parentheses.

We also find that firms headquartered in an industry with more SOEs will contribute both more funds and materials toward helping the poor. The coefficient of *SOE ratio* in column 2 is 4.809 (p<0.01); the value is about twice as much as that in column 4 (γ = 2.345, p<0.05). The table also shows that industry *HHI* is not associated with a firm’s funding or material contribution to poverty alleviation, which is consistent with the empirical result in Table 6.

Column 6 of Table 6 demonstrates that the coefficients of *Gov_Intervention*, *Poverty rate*, and *GDP per capita* are 1.133 (p<0.05), 0.152 (p<0.1), and -1.929 (p<0.05), respectively. Column 8 of Table 6 presents that the coefficients of *Gov_Intervention*, *Poverty rate*, and *GDP per capita* are respectively 0.106 (p<0.01), 0.120 (p<0.1), and -2.345 (p<0.01). These results suggest that firms headquartered in a province that is less prosperous, has higher poverty incidence, and has stronger government intervention are more likely to make monetary and material contributions for the anti-poverty campaign. This once again supports H4 to H6, although H5 is weakly supported.

## Conclusions, implications and directions for future research

This study examines the external environment factors on a firm’s willingness toward and contribution to China’s anti-poverty drive using a multilevel model. We use data of listed firms that have disclosed poverty alleviation works in China’s Shanghai and Shenzhen stock markets from 2016 to 2019. The findings are as follows. (1) Whether firms compete in a competitive industry or not, their will, behavior, and plans will not be swayed. (2) For more dynamic industries, firms are less likely to make substantial contributions and invest less for poverty alleviation. (3) In an industry with more SOEs, firms not only are more inclined to participate in poverty alleviation, but they also make more substantial contributions to poor areas. (4) Firms headquartered in a province with more prosperous or lesser government intervention are less likely to contribute to poverty alleviation, especially in terms of a substantial contribution (no matter for willingness or intensity). (5) Firms headquartered in a province with a higher poverty rate do not really show a difference in their willingness to participate in poverty alleviation activities and to make substantial contributions, although they are associated with more investment in the anti-poverty campaign. Our additional research discovers that firms competitive in an industry under a less dynamic environment are more likely to invest funds into poverty alleviation, while industry dynamism is not associated with material contribution. Firms headquartered in an industry with more SOEs and in a province with a stronger government, higher poverty rate, and lower per capita GDP are more likely to make both monetary and material contributions towards the anti-poverty campaign.

Our research indicates that a firm’s willingness and contribution toward helping with the societal goal of poverty eradication are shaped by industry dynamism, poverty alleviation pattern in industry (i.e., number of SOEs that might take the lead), government intervention, and regional economic climate. This provides some important practical implications for authorities on the successful accomplishment of a broad poverty reduction goal. First, modern corporations are rational. When they face uncertainty and dynamism, their primary decision is to apply limited resources and cash flow into profitable instead of charitable activities. Thus, governments should provide a win-win solution to appeal to firms to assist them in solving social problems. Second, SOEs’ strategies and behaviors generate a pattern within an industry. Thus, the government can select and promote prosocial individuals to serve as executives of SOEs due to the appointment right by the upper-level Organization Department of the Chinese Communist Party. If SOEs positively respond to state strategies and government requirements, then private companies in the industry are likely to treat them as benchmarks and imitate them in their own prosocial activities. This is the peer effect within the industry. Third, our results show that the government can influence corporate contributions on poverty alleviation by putting legitimacy pressure on firms. Government influence is strong and particularly favorable to institutionally-driven behavior. Therefore, governments can properly use their power to encourage more corporate participation in solving social problems such as poverty eradication, rural revitalization, and economic development. Finally, we note that the economic climate and poverty rate is associated with firms’ likelihood and their extent to take part in poverty reduction. This reflects that many enterprises follow local economic conditions to decide whether to participate in poverty reduction or not. Though that is inconsistent with the view of local governments and local enterprises, it would be better to encourage firms to cultivate a global perspective and to spare some concentration of resources on the needs of their own country.

Our paper also paves the way for several potential avenues of future research. Firstly, our investigation into the factors influencing a firm’s willingness and contribution to China’s anti-poverty efforts is presently confined to the external environment. However, existing literature suggests that internal factors within a firm, such as its characteristics, CSR initiatives and motivations, executives’ political connections and experiences with poverty [4,12,25], also play a crucial role in determining a firm’s contribution to poverty alleviation. Exploring the interplay of both external and internal factors in corporations’ engagement with poverty alleviation activities could be a valuable direction for future inquiry. Secondly, our paper primarily focuses on quantifying the amounts of corporate contributions, without delving into the specifics of the projects in which firms are involved or assessing the effectiveness of these initiatives. Incorporating qualitative data from corporations’ poverty reduction reports to complement the quantitative information utilized in our study holds the potential to significantly enhance our comprehension of the private sector’s impact on poverty alleviation. This multifaceted approach could provide a more comprehensive insight into the strategies and outcomes of corporate anti-poverty endeavors. Lastly, our study is firmly situated within the Chinese context, reveals how diverse industries and provinces influence firms’ inclination for poverty alleviation, making the direct extension of our findings to other developed markets less straightforward. Moreover, national economic, market, and political environment influence corporate philanthropic and social responsibility initiatives. Hence, conducting a comparative analysis using cross-country samples has the potential to offer a more holistic perspective and deepen our understanding of corporations’ attitudes and efforts towards anti-poverty drive.

## References

[1] Bruton GD, Ketchen DJ, Ireland RD. Entrepreneurship as a solution to poverty. J Bus Ventur. 2013;28: 683–689. doi: 10.1016/j.jbusvent.2013.05.002

[2] Prahalad CK. Promises and perils at the bottom of the pyramid. Adm Sci Q. 2005;50: 504–523.

[3] Bruton GD, Ahlstrom D, Si S. Entrepreneurship, poverty, and Asia: Moving beyond subsistence entrepreneurship. Asia Pac J Manag. 2015;32: 1–22. doi: 10.1007/s10490-014-9404-x

[4] Chang Y, He W, Wang J. Government Initiated Corporate Social Responsibility Activities: Evidence from a Poverty Alleviation Campaign in China. J Bus Ethics. 2021;173: 661–685. doi: 10.1007/s10551-020-04538-w

[5] Qu T. Poverty alleviation in China–plan and action. China J Soc Work. 2017;10: 79–85. doi: 10.1080/17525098.2017.1314812

[6] Okpara JO, Wynn PM. Stakeholders’ perceptions about corporate social responsibility: Implications for poverty alleviation. Thunderbird Int Bus Rev. 2012;54: 13.

[7] Lee M-DP. Configuration of External Influences: The Combined Effects of Institutions and Stakeholders on Corporate Social Responsibility Strategies. J Bus Ethics. 2011;102: 281–298. doi: 10.1007/s10551-011-0814-0

[8] Aggarwal VS, Jha A. Pressures of CSR in India: an institutional perspective. J Strategy Manag. 2019;12: 227–242. doi: 10.1108/JSMA-10-2018-0110

[9] Matten D, Moon J. “Implicit” and “Explicit” CSR: A Conceptual Framework for a Comparative Understanding of Corporate Social Responsibility. Acad Manage Rev. 2008;33: 404–424. doi: 10.5465/AMR.2008.31193458

[10] Vashchenko M. An external perspective on CSR: What matters and what does not? Bus Ethics Eur Rev. 2017;26. doi: 10.1111/beer.12162

[11] Marquis C, Glynn MA, Davis GF. Community Isomorphism and Corporate Social Action. Acad Manage Rev. 2007;32: 925–945. doi: 10.5465/AMR.2007.25275683

[12] Amato LH, Amato CH. The Effects of Firm Size and Industry on Corporate Giving. J Bus Ethics. 2007;72: 229–241. doi: 10.2307/25075376

[13] Luo J, Xiang Y, Zhu R. Military top executives and corporate philanthropy: Evidence from China. Asia Pac J Manag. 2017;34: 725–755. doi: 10.1007/s10490-016-9499-3

[14] Muller A, Whiteman G. Exploring the Geography of Corporate Philanthropic Disaster Response: A Study of Fortune Global 500 Firms. J Bus Ethics. 2009;84: 589–603. doi: 10.1007/s10551-008-9710-7

[15] Gao Y, Yang H, Lin YL. What’s the Value in It? Corporate Philanthropic Giving under Uncertainty. Asia Pac J Manag. 2017;34: 215–240. doi: 10.1007/s10490-016-9478-8

[16] Zhang J, Marquis C, Qiao K. Do Political Connections Buffer Firms from or Bind Firms to the Government? A Study of Corporate Charitable Donations of Chinese Firms. Organ Sci. 2016;27: 1307–1324. doi: 10.1287/orsc.2016.1084

[17] Marshall N, Dawley S, Pike A, Pollard J. Geographies of corporate philanthropy: The Northern Rock Foundation. Environ Plan. 2018;50: 266–287. doi: 10.1177/0308518X17746405

[18] McWilliams A, Siegel D. Corporate Social Responsibility: A Theory of the Firm Perspective. Acad Manage Rev. 2001;26: 117–127. doi: 10.2307/259398

[19] Carroll AB. A Three-Dimensional Conceptual Model of Corporate Performance. Acad Manage Rev. 1979;4: 497–505. doi: 10.5465/amr.1979.4498296

[20] Gautier A, Pache A-C. Research on Corporate Philanthropy: A Review and Assessment. J Bus Ethics. 2015;126: 343–369. doi: 10.1007/s10551-013-1969-7

[21] Aguinis H, Glavas A. What We Know and Don’t Know About Corporate Social Responsibility. J Manag. 2012;38: 932–968. doi: 10.1177/0149206311436079

[22] Yin J, Singhapakdi A, Du Y. Causes and moderators of corporate social responsibility in China: The influence of personal values and institutional logics. Asian Bus Manag. 2016;15: 226–254. doi: 10.1057/s41291-016-0001-3

[23] Cha W, Rajadhyaksha U. What do we know about corporate philanthropy? A review and research directions. Bus Ethics Eur Rev. 2021; 1–25. doi: 10.1111/beer.12341

[24] Salah W. The Impact of Country-Level and Firm-Level on Financial Performance: A Multilevel Approach. Int J Account Tax. 2018;6. doi: 10.15640/ijat.v6n2a5

[25] Medina‐Muñoz RD, Medina‐Muñoz DR. Corporate social responsibility for poverty alleviation: An integrated research framework. Bus Ethics Eur Rev. 2020;29: 3–19. doi: 10.1111/beer.12248

[26] Zhang R, Zhu J, Yue H, Zhu C. Corporate Philanthropic Giving, Advertising Intensity, and Industry Competition Level. J Bus Ethics. 2010;94: 39–52. doi: 10.1007/s10551-009-0248-0

[27] Zhang C, Liu Q, Ge G, Hao Y, Hao H. The impact of government intervention on corporate environmental performance: Evidence from China’s national civilized city award. Finance Res Lett. 2021;39: 101624. doi: 10.1016/j.frl.2020.101624

[28] Singh S, Guha M. Peer Effect on Corporate Social Responsibility: Investigating Moderating Role of Business Group Affiliation, State Ownership, and Firm Size. Int J Strateg Decis Sci. 2019;10: 114–130. doi: 10.4018/IJSDS.2019070107

[29] Lubatkin MH, Simsek Z, Ling Y, Veiga JF. Ambidexterity and Performance in Small-to Medium-Sized Firms: The Pivotal Role of Top Management Team Behavioral Integration. J Manag. 2006;32: 646–672. doi: 10.1177/0149206306290712

[30] Roberts RW. Determinants of corporate social responsibility disclosure: An application of stakeholder theory. Account Organ Soc. 1992;17: 595–612. doi: 10.1016/0361-3682(92)90015-K

[31] McGuire JB, Sundgren A, Schneeweis T. Corporate Social Responsibility and Firm Financial Performance. Acad Manage J. 1988;31: 854–872. doi: 10.5465/256342

[32] Jha A, Cox J. Corporate social responsibility and social capital. J Bank Finance. 2015;60: 252–270. doi: 10.1016/j.jbankfin.2015.08.003

[33] Li W, Zhang R. Corporate Social Responsibility, Ownership Structure, and Political Interference: Evidence from China. J Bus Ethics. 2010;96: 631–645. doi: 10.1007/s10551-010-0488-z

[34] Kim H-D, Kim T, Kim Y, Park K. Do long-term institutional investors promote corporate social responsibility activities? J Bank Finance. 2019;101: 256–269. doi: 10.1016/j.jbankfin.2018.11.015

[35] Metzger L, Nunnenkamp P, Mahmoud TO. Is Corporate Aid Targeted to Poor and Deserving Countries? A Case Study of Nestlé’s Aid Allocation. World Dev. 2010;38: 228–243. doi: 10.1016/j.worlddev.2009.09.005

[36] Husted BW, Jamali D, Saffar W. Near and dear? The role of location in CSR engagement. Strateg Manag J. 2016;37: 2050–2070. doi: 10.1002/smj.2437

[37] Oliver C. Strategic Responses to Institutional Processes. Acad Manage Rev. 1991;16: 145–179. doi: 10.5465/amr.1991.4279002

[38] Hess D, Warren DE. The Meaning and Meaningfulness of Corporate Social Initiatives. Bus Soc Rev. 2008;113: 163–197. doi: 10.1111/j.1467-8594.2008.00317.x

[39] Idemudia U. Corporate social responsibility and developing countries: moving the critical CSR research agenda in Africa forward. Prog Dev Stud. 2011;11: 1–18. doi: 10.1177/146499341001100101

[40] Johnson O. Corporate Philanthropy: An Analysis of Corporate Contributions. J Bus. 1966;39: 489–504. doi: 10.1086/294890

[41] Useem M. Market and institutional factors in corporate contributions. Calif Manage Rev. 1988;30. doi: 10.2307/41166548

[42] Kolk A, van Tulder R. Poverty alleviation as business strategy? Evaluating commitments of frontrunner Multinational Corporations. World Dev. 2006;34: 789–801. doi: 10.1016/j.worlddev.2005.10.005

[43] Gao Y, Hafsi T. Government Intervention, Peers’ Giving and Corporate Philanthropy: Evidence from Chinese Private SMEs. J Bus Ethics. 2015;132: 433–447. doi: 10.1007/s10551-014-2329-y

[44] Cao J, Liang H, Zhan X. Peer Effects of Corporate Social Responsibility. Manag Sci. 2019; mnsc.2018.3100. doi: 10.1287/mnsc.2018.3100

[45] Marquis C, Tilcsik A. Institutional Equivalence: How Industry and Community Peers Influence Corporate Philanthropy. Organ Sci. 2016;27: 1325–1341. doi: 10.1287/orsc.2016.1083

[46] Chen Z, Fuller DB, Zheng L. Institutional isomorphism and Chinese private corporate philanthropy: state coercion, corruption, and other institutional effects. Asian Bus Manag. 2018;17: 83–111. doi: 10.1057/s41291-018-0032-z

[47] Gao Y. Philanthropic disaster relief giving as a response to institutional pressure: Evidence from China. J Bus Res. 2011;64: 1377–1382. doi: 10.1016/j.jbusres.2010.12.003 32287523 PMC7132407

[48] Yang Y, Tang M. Finding the Ethics of “Red Capitalists”: Political Connection and Philanthropy of Chinese Private Entrepreneurs. J Bus Ethics. 2020;161: 133–147. doi: 10.1007/s10551-018-3934-y

[49] Li S, Song X, Wu H. Political Connection, Ownership Structure, and Corporate Philanthropy in China: A Strategic-Political Perspective. J Bus Ethics. 2015;129: 399–411. doi: 10.1007/s10551-014-2167-y

[50] Chen X, Wan P. Social trust and corporate social responsibility: Evidence from China. Corp Soc Responsib Environ Manag. 2020;27: 485–500. doi: 10.1002/csr.1814

[51] Dong M, Stettler A. Estimating firm-level and country-level effects in cross-sectional analyses: An application of hierarchical modeling in corporate disclosure studies. Int J Account. 2011;46: 271–303. doi: 10.1016/j.intacc.2011.07.002

[52] Fernández-Kranz D, Santaló J. When Necessity Becomes a Virtue: The Effect of Product Market Competition on Corporate Social Responsibility. J Econ Manag Strategy. 2010;19: 453–487. doi: 10.1111/j.1530-9134.2010.00258.x

[53] Wang Q, Wong TJ, Xia L. State ownership, the institutional environment, and auditor choice: Evidence from China. J Account Econ. 2008;46: 112–134. doi: 10.1016/j.jacceco.2008.04.001

[54] Jeong Y-C, Kim T-Y. Between Legitimacy and Efficiency: An Institutional Theory of Corporate Giving. Acad Manage J. 2019;62: 1583–1608. doi: 10.5465/amj.2016.0575

[55] Aragón-Correa JA, Sharma S. A Contingent Resource-Based View of Proactive Corporate Environmental Strategy. Acad Manage Rev. 2003;28: 71–88. doi: 10.5465/amr.2003.8925233

[56] Lewis GJ, Harvey B. Perceived Environmental Uncertainty: The Extension of Miller’s Scale to the Natural Environment. J Manag Stud. 2001;38: 201–234. doi: 10.1111/1467-6486.00234

[57] Milliken FJ. Three Types of Perceived Uncertainty About the Environment: State, Effect, and Response Uncertainty. Acad Manage Rev. 1987 [cited 12 Sep 2022]. doi: 10.5465/amr.1987.4306502

[58] Mitchell JR, Shepherd DA, Sharfman MP. Erratic strategic decisions: when and why managers are inconsistent in strategic decision making. Strateg Manag J. 2011;32: 683–704. doi: 10.1002/smj.905

[59] Jansen JJP, Van Den Bosch FAJ, Volberda HW. Exploratory Innovation, Exploitative Innovation, and Performance: Effects of Organizational Antecedents and Environmental Moderators. Manag Sci. 2006;52: 1661–1674. doi: 10.1287/mnsc.1060.0576

[60] Sun W, Price JM. The impact of environmental uncertainty on increasing customer satisfaction through corporate social responsibility. Eur J Mark. 2016;50: 1209–1238. doi: 10.1108/EJM-02-2015-0077

[61] Zhang Z, Hu D, Liang L. The impact of supplier dependence on suppliers’ CSR: The moderating role of industrial dynamism and corporate transparency. J Purch Supply Manag. 2021;27: 100702. doi: 10.1016/j.pursup.2021.100702

[62] Miller D, Friesen PH. Innovation in conservative and entrepreneurial firms: Two models of strategic momentum. Strateg Manag J. 1982;3: 1–25. doi: 10.1002/smj.4250030102

[63] Li Y, Dai J, Cui L. The impact of digital technologies on economic and environmental performance in the context of industry 4.0: A moderated mediation model. Int J Prod Econ. 2020;229: 107777. doi: 10.1016/j.ijpe.2020.107777

[64] Chen H, Zeng S, Lin H, Ma H. Munificence, Dynamism, and Complexity: How Industry Context Drives Corporate Sustainability. Bus Strategy Environ. 2017;26: 125–141. doi: 10.1002/bse.1902

[65] Gao Y, Lin YL, Yang H. What’s the value in it? Corporate giving under uncertainty. Asia Pac J Manag. 2017;34: 215–240. doi: 10.1007/s10490-016-9478-8

[66] Palmer TB, Wiseman RM. Decoupling risk taking from income stream uncertainty: a holistic model of risk. Strateg Manag J. 1999;20: 1037–1062. doi: 10.1002/(SICI)1097-0266(199911)20:11&lt;1037::AID-SMJ67&gt;3.0.CO;2–2

[67] Lewis BW, Walls JL, Dowell GWS. Difference in degrees: CEO characteristics and firm environmental disclosure. Strateg Manag J. 2014;35: 712–722. doi: 10.1002/smj.2127

[68] Goll I, Rasheed AA. The Moderating Effect of Environmental Munificence and Dynamism on the Relationship Between Discretionary Social Responsibility and Firm Performance. J Bus Ethics. 2004;49: 41–54. doi: 10.1023/B:BUSI.0000013862.14941.4e.

[69] Li J, Tang Y. CEO Hubris and Firm Risk Taking in China: The Moderating Role of Managerial Discretion. Acad Manage J. 2010;53: 45–68. doi: 10.5465/amj.2010.48036912

[70] Ghosh D, Olsen L. Environmental uncertainty and managers’ use of discretionary accruals. Account Organ Soc. 2009;34: 188–205. doi: 10.1016/j.aos.2008.07.001

[71] Patel PC, Pearce JA, Oghazi P. Not so myopic: Investors lowering short-term growth expectations under high industry ESG-sales-related dynamism and predictability. J Bus Res. 2020. doi: 10.1016/j.jbusres.2020.11.013

[72] Chen H, Guo Y, Wen Q. For goodwill or resources? The rationale behind firms’ corporate philanthropy in an environment with high economic policy uncertainty. China Econ Rev. 2021;65: 101580. doi: 10.1016/j.chieco.2020.101580

[73] Phan HV, Nguyen NH, Nguyen HT, Hegde S. Policy uncertainty and firm cash holdings. J Bus Res. 2019;95: 71–82. doi: 10.1016/j.jbusres.2018.10.001

[74] Donaldson T, Preston LE. The Stakeholder Theory of the Corporation: Concepts, Evidence, and Implications. Acad Manage Rev. 1995;20: 65–91. doi: 10.5465/amr.1995.9503271992

[75] Du X, Jian W, Du Y, Feng W, Zeng Q. Religion, the Nature of Ultimate Owner, and Corporate Philanthropic Giving: Evidence from China. J Bus Ethics. 2014;123: 235–256. doi: 10.1007/s10551-013-1804-1

[76] Kaustia M, Rantala V. Social learning and corporate peer effects. J Financ Econ. 2015;117: 653–669. doi: 10.1016/j.jfineco.2015.06.006

[77] Liu S, Wu D. Competing by conducting good deeds: The peer effect of corporate social responsibility. Finance Res Lett. 2016;16: 47–54. doi: 10.1016/j.frl.2015.10.013

[78] Suchman MC. Managing Legitimacy: Strategic and Institutional Approaches. Acad Manage Rev. 1995;20: 571–610. doi: 10.5465/amr.1995.9508080331

[79] Makino S, Isobe T, Chan CM. Does country matter? Strateg Manag J. 2004;25: 1027–1043. doi: 10.1002/smj.412

[80] Tong TW, Alessandri TM, Reuer JJ, Chintakananda A. How much does country matter? An analysis of firms’ growth options. J Int Bus Stud. 2008;39: 387–405. doi: 10.1057/palgrave.jibs.8400355

[81] Sheng S, Zhou KZ, Li JJ. The Effects of Business and Political Ties on Firm Performance: Evidence from China. J Mark. 2011;75: 1–15. doi: 10.1509/jm.75.1.1

[82] Xu C. The Fundamental Institutions of China’s Reforms and Development. J Econ Lit. 2011;49: 1076–1151. doi: 10.1257/jel.49.4.1076

[83] Kong G, Kong TD, Lu R. Political promotion incentives and within-firm pay gap: Evidence from China. J Account Public Policy. 2020;39: 106715. doi: 10.1016/j.jaccpubpol.2020.106715

[84] Deng L, Jiang P, Li S, Liao M. Government intervention and firm investment. J Corp Finance. 2020;63: 101231. doi: 10.1016/j.jcorpfin.2017.07.002

[85] Feng L, Fu T, Apergis N, Tao H, Yan W. The role of government intervention in financial development: micro-evidence from China. Account Finance. 2019;59: 2855–2878. doi: 10.1111/acfi.12559

[86] Chen D, Khan S, Yu X, Zhang Z. Government Intervention and Investment Comovement: Chinese Evidence. J Bus Finance Account. 2013;40: 564–587. doi: 10.1111/jbfa.12022

[87] Hao Y, Lu J. The Impact of Government Intervention on Corporate Investment Allocations and Efficiency: Evidence from China. Financ Manag. 2018;47: 383–419. doi: 10.1111/fima.12188

[88] Cull R, Xu LC, Yang X, Zhou L-A, Zhu T. Market facilitation by local government and firm efficiency: Evidence from China. J Corp Finance. 2017;42: 460–480. doi: 10.1016/j.jcorpfin.2015.06.002

[89] Bhattacharya U, Hsu P-H, Tian X, Xu Y. What Affects Innovation More: Policy or Policy Uncertainty? J Financ Quant Anal. 2017;52: 1869–1901. doi: 10.1017/S0022109017000540

[90] Campbell JL. Why would corporations behave in socially responsible ways? an institutional theory of corporate social responsibility. Acad Manage Rev. 2007;32: 946–967. doi: 10.5465/amr.2007.25275684

[91] Besley T, Burgess R. Halving global poverty. J Econ Perspect. 2003;17: 3–22. doi: 10.1257/089533003769204335

[92] Marrero GA, Servén L. Growth, inequality and poverty: a robust relationship? Empir Econ. 2022;63: 725–791. doi: 10.1007/s00181-021-02152-x 34840407 PMC8610444

[93] Wang XL, Fan G, Hu LP. Marketization Index of China’s Provinces. Beijing: Social Sciences Academic Press; 2018.

[94] Long C, Yang J. What explains Chinese private entrepreneurs’ charitable behaviors?—A story of dynamic reciprocal relationship between firms and the government. China Econ Rev. 2016;40: 1–16. doi: 10.1016/j.chieco.2016.05.001

[95] Short JC, McKelvie A, Ketchen DJ, Chandler GN. Firm and industry effects on firm performance: A generalization and extension for new ventures: New Venture Performance. Strateg Entrep J. 2009;3: 47–65. doi: 10.1002/sej.53

[96] Peterson RS, Behfar KJ. The dynamic relationship between performance feedback, trust, and conflict in groups: A longitudinal study. Organ Behav Hum Decis Process. 2003;92: 102–112. doi: 10.1016/S0749-5978(03)00090-6

[97] Aguinis H, Gottfredson RK, Culpepper SA. Best-Practice Recommendations for Estimating Cross-Level Interaction Effects Using Multilevel Modeling. J Manag. 2013;39: 1490–1528. doi: 10.1177/0149206313478188

[98] Slade Shantz A, Kistruck G, Zietsma C. The opportunity not taken: The occupational identity of entrepreneurs in contexts of poverty. J Bus Ventur. 2018;33: 416–437. doi: 10.1016/j.jbusvent.2018.02.003

[99] D’agostino RB, Belanger A, D’agostino RB. A Suggestion for Using Powerful and Informative Tests of Normality. Am Stat. 1990;44: 316–321. doi: 10.1080/00031305.1990.10475751

[100] Du X. Is Corporate Philanthropy Used as Environmental Misconduct Dressing? Evidence from Chinese Family-Owned Firms. J Bus Ethics. 2015;129: 341–361. doi: 10.1007/s10551-014-2163-2

